# A Case of Severe Uterine Hemorrhage, Two Weeks after Delivery

**Published:** 1882-12-20

**Authors:** T. B. Greenley

**Affiliations:** Kentucky


### Southern Medical Record:

EDITORS:

T. S. POWELL, M.D. W. T. GOLDSMITH, M.D. R. C. WORD, M.D.

*R. C. WORD, M.D., Managing Editor.*

☞All Communications and Letters on Business connected with the RECORD must be addressed to the Managing Editor.

VOL. XII. ATLANTA, GA., DEC. 20, 1882. No. 12.

### ORIGINAL AND SELECTED ARTICLES.

### A CASE OF SEVERE UTERINE HEMORRHAGE—TWO WEEKS AFTER DELIVERY.

BY T. B. GREENLEY, M. D., OF KENTUCKY.

Mrs. C., aged 28 years, of good general health and family record, was confined on August 3d, 1881. The labor was natural in every particular, and she expressed herself as having a better time than either of her previous labors, this being her third. Her lying-in period was attended with no untoward symptom; her lochial discharge somewhat diminished in quantity; her appetite good, and feeling so well at the expiration of two weeks she got up and was about the house and out in the yard. While in the yard, without any premonition, she was taken suddenly with a severe hemorrhage from the womb, and by the time she was taken into the house and put to bed she was pulseless. By the time I arrived, about an hour and a half after the attack, the bleeding had measurably ceased, but she was still nearly pulseless. Her mother, who was present, had the thought to lower her head by elevating the foot of the bed, which probably had the effect of partially restoring the failing action of the heart.

By the use of proper stimulants and hemostatics, I soon succeeded in arresting the farther flow of blood and establishing reaction.

I saw her every day for one week, and by the use of proper kinds of nourishment—the principal part of which was milk, she rallied and felt strong enough to sit up, and expressed a desire to do so, but I cautioned her that it would not be safe to do so for several days yet. When I left her on this day I told her she was doing so well that I would not return unless sent for. But the night following, a messenger came after me in a great hurry, stating that Mrs. C. was dying from a second hemorrhage. I hastened to her bedside, and found her pulseless and completely pallid. I now thought she would certainly die before reaction could be brought about. On examination found blood still oozing from the womb. Used tampons, and exhibited ergot, stimulants and nourishment, as well as warmth, etc., to the extremities. It was nearly twenty-four hours before reaction was established, so that the pulse could be felt at wrist. At this critical period I had my friend, Professor Balling, of Louisville, sent for. In order to arrest the oozing from the womb we injected tincture iodine and carbolic acid into the organ, which had the desired effect. She now improved gradually until Monday night, the 29th, when she had a very severe chill, followed by high fever. The second hemorrhage occurred on Wednesday, the 23d. She had had no excess of temperature from the time of the first flooding on the 27th up to the time of the chill on night of the 29th; but after the chill her temperature was 104.5. The fever continued high until next evening (30th) when it gradually declined until 1 o'clock, when there was only one degree excess of heat. About daylight on the morning of the 31st, she had a second chill, with as high reaction as followed the first.

Her temperature again fell within one degree of normal in the evening (Wednesday) and she had no more chills. There was some fever during Thursday, September 1st, and on Friday, the 2d, gradually became higher until the next day—her temperature again reached 104.5; but by midnight fell to 88.5.

Next morning, Sunday, September 4th, her fever began to rise, and by 4 o'clock attained its maximum, 104.5. The same occurred on Monday, the 5th, but on Tuesday, the 6th, her temperature did not get higher than 103.5 in the afternoon, and gradually abating until midnight, when it was 100.5.

Wednesday, the 7th, her temperature again rose to 103.5, and at night went down to 101. On Thursday morning her temperature again came up to 103.5 She died on this day at 10 o'clock A. M.

My friend and neighbor, Dr. Foss, saw the patient with me on the 2d and 7th of September, the last visit being on the day before she died.

The treatment of this case, after fever was developed, consisted mainly in the use of quinine and nourishment, together with the solution of hyposulphite soda. On account of the irritability of the stomach we could not use full doses of quinine, but used some by enema, but diarrhœa, which supervened soon after the fever came on, prevented, to some extent, the good effects of the remedy administered in this way. There was more or less delirium after supervention of fever.

In studying the history of this case, two questions naturally present themselves to the mind of the reader: First, what caused the hemorrhage? and secondly, what caused the fever?

Some might say getting up too soon after delivery was the cause of the flowing; but I cannot take this view of the case, as I have never known a woman to have flooding two weeks after child-birth when everything attending her confinement was natural, and the lochial dicharge having disappeared, as was the case with this patient. As to the second question, some might say the chills and subsequent fever were possibly due to peritonitis. But we are compelled to exclude this view, from the fact there was no tenderness, pain or tympanitis. Then what could have caused the hemorrhage and fever?

My theory of the cause of both is "malaria." After the fever was developed I began to look around the premises to discover the cause, if possible. I found that the house, which was very old and made of logs, had settled, on account of the ground sills having rotted, until the floor had reached the dirt, and the sleepers buried. The house was not guttered, and eave water settled under the house, and could not dry out. The patient had made the lower room over this floor her sleeping apartment for some two or three weeks before her confinement, and continued to do so afterwards.

Typho-malarial fever, accompanied or preceded by hemorrhage, is not very common in this latitude, although I have had several cases of nasal and enteric hemorrhage accompanying this fever. But the question might be asked, could malarial impress act as the exciting cause of hemorrhage previous to the development of the fever? In this case it will be observed that the flooding preceded the fever by some twelve days.

The only way I can see how malarial impress could produce or act as a cause of hemorrhage is by lowering the vital forces and thereby producing attenuation of the blood. There is but little doubt that malaria has these effects on the system prior to the development of fever; and if so, why may not hemorrhage result before as well as after fever is manifested?

The reason I am so particular about reporting this case is the fact that the patient had her life insured in the Presbyterian Benefit Association of Louisville, there being a provision in the policy that if a woman dies from the effects of child-birth the Association is exempt from payment.

The question arose in the mind of the medical directors of the institution whether or not the patient died from the effects of labor; but on the judgments of Dr. Foss and myself the policy was paid.

Dr. Balling did not see the patient after the fever made its appearance, and could not give an opinion.

